# Nuclear and Mitochondrial DNA Alterations in Newborns with Prenatal Exposure to Cigarette Smoke

**DOI:** 10.3390/ijerph120201135

**Published:** 2015-01-22

**Authors:** Francesca Pirini, Elisa Guida, Fahcina Lawson, Andrea Mancinelli, Rafael Guerrero-Preston

**Affiliations:** 1 Head and Neck Cancer Research Division, Otolaryngology Department, School of Medicine, The Johns Hopkins University, Baltimore, MD 21205, USA; E-Mails: francesca.pirini@gmail.com (F.P.); eguida1@jhmi.edu (E.G.); flawson1@jhmi.edu (F.L.); andrea.mancinelli@gmail.com (A.M.); 2 Department of Obstetrics and Gynecology, School of Medicine, The University of Puerto Rico, San Juan 00936, Puerto Rico

**Keywords:** maternal cigarette smoke effects, epigenomics effects, mitochondrial DNA, *in utero* exposures, DNA methylation, environmental cigarette smoke effects, molecular biomarkers

## Abstract

Newborns exposed to maternal cigarette smoke (CS) *in utero* have an increased risk of developing chronic diseases, cancer, and acquiring decreased cognitive function in adulthood. Although the literature reports many deleterious effects associated with maternal cigarette smoking on the fetus, the molecular alterations and mechanisms of action are not yet clear. Smoking may act directly on nuclear DNA by inducing mutations or epigenetic modifications. Recent studies also indicate that smoking may act on mitochondrial DNA by inducing a change in the number of copies to make up for the damage caused by smoking on the respiratory chain and lack of energy. In addition, individual genetic susceptibility plays a significant role in determining the effects of smoking during development. Furthermore, prior exposure of paternal and maternal gametes to cigarette smoke may affect the health of the developing individual, not only the *in utero* exposure. This review examines the genetic and epigenetic alterations in nuclear and mitochondrial DNA associated with smoke exposure during the most sensitive periods of development (prior to conception, prenatal and early postnatal) and assesses how such changes may have consequences for both fetal growth and development.

## 1. Introduction

The disease risks from cigarette smoking increased over most of the 20th century [1]. As a matter of fact, direct cigarette smoke exposure cause six million deaths each year and more than 600,000 are associated with second-hand smoke all over the world [2]. Interestingly, the health consequences of tobacco smoke are particularly severe during critical periods of life, for instance smoking during pregnancy is the most important risk factor for adverse pregnancy outcomes [3].

Cigarette smoke, with its mix of hazardous chemicals, carcinogens, pulmonary irritants, cilia toxicants, cardiotoxin and teratogens, has been associated with childhood diseases and lifelong risks of chronic disease [4,5] pre-term birth [6], asthma [7], decreased cognitive function [8] and infertility [9]. There are three sensitive periods during the very early stage of development where a disruption or modification of the environment can influence fetal and offspring development: pre-conception, *in utero* and perinatal. Even if the two latter periods have been most extensively studied, it is well known that maternal lifestyle and environmental exposures during these three periods can lead to adverse health effects throughout the life course. For instance, *in utero* exposure to cigarette smoke is associated with reduced fecundity in females and with altered reproductive development in males [10] and cigarette exposure during the early postnatal period is linked to higher frequency of chronic diseases during the life-course [11]. Regarding the first time-window, the pre-conceptional tobacco exposure effects (genomic instability and DNA damage) in the offspring haven’t been widely characterized in humans because of the difficulty of discerning between pre- and post-conception effects, but interesting studies in rodent models have confirmed them [12,13].

The critical role of early life events that increase susceptibility to certain chronic diseases resides in the developmental plasticity feature of mammalian organisms. This plasticity consists in the modulation of gene expression during early development by environmental and intrinsic characteristics. Developmental plasticity is mediated, at least in part, by epigenetic processes such as DNA methylation and histone modifications that lead to changes in gene expression [14]. Therefore, even if the mechanisms of action are not completely characterized, tobacco smoke exposure during early human development, similarly to other environmental cues, affects the mature phenotype of an organism by altering both the genome and the epigenome [15,16]. Moreover, recent studies also suggest that the mitochondrial machinery is also involved [17,18].

In this review, we analyze the genetic and epigenetic alterations in nuclear and mitochondrial DNA associated with smoke exposure during the prenatal and early postnatal periods of development, covering the most crucial findings and revising the literature related to these critical epigenetic modifications.

## 2. Genotoxic Effects on Nuclear DNA

Exposures to environmental contaminants and lifestyle choices during gestation and/or shortly after birth can significantly influence individual phenotype and susceptibility to disease during childhood and throughout the life course. Moreover, environmental exposures also represent the major source of mutation and DNA damage that threaten the integrity of the genome, at gene and chromosome levels.

The mutagenic impact of maternal cigarette smoking on fetal DNA has been described in several studies [19]. In adults the exposure to tobacco causes a 10%–20% increase in chromosome aberrations frequency [20], result confirmed by Pluth and colleagues in newborns subject to *in utero* exposure [21]. In an *in vitro* study on fetal cells (amniocytes) treated with nicotine, Demirhan and colleagues found an increase in chromosomal aberrations (CAs) in cells exposed to nicotine [22]. In vivo, the chromosomal instability in amniocytes correlates with the number of cigarettes as well as with chromosomal translocations, DNA damage, mutations and micronucleation in cord blood [23,24,25]. Another study shows that *in utero* exposure to cigarette smoke or environmental tobacco smoke (second-hand smoke) led to a significant increase in DNA damage. They also reported a direct association between subject with serious DNA damage and low birth weight of neonates, an indicator of health problems later in adult life [26].

Therefore, there is a large body of evidence that demonstrates the direct genotoxic effects of smoke components, namely nicotine and its metabolized derivatives, all along the life course, as well as during embryonic development.

## 3. Individual Susceptibility

Not all mothers who smoke have children with consequences such as low birth weight or intrauterine growth restriction. The reason for this variability is largely unknown, but it may be related to individual genetic susceptibility. Studies that examine genetic polymorphisms associated to tobacco smoke exposure reveal variability in the frequency of single nucleotide polymorphysms (SNPs) in genes associated with the metabolism of polycyclic aromatic hydrocarbons (PAHs) and aromatic amines. The genes with the highest frequency of SNPs associated to cigarette smoke exposure are isoenzyme of cytochrome P450 (*CYP1A1*)*,* glutathione S-transferase (*GST*), and N-acetyltransferases (*NAT*) [27,28,29,30,31]. In order to interact with DNA and exert their genotoxic potential, multiple tobacco carcinogens require metabolic activation by phase I enzymes (e.g., *CYP1A1*), while phase II enzymes, including *NAT2*, *GSTM1* and *GSTT1*, catalyze the activation or deactivation of aromatic and heterocyclic amine carcinogens and intermediate products generated by the *CYP1A1* enzyme. Each of these enzymes exhibits SNPs that have been shown to be related to adverse pregnancy outcomes and specific cancers [26,27,28]. Wang and colleagues [27] showed how maternal *CYP1A1* and *GSTT1* genotypes modified the association between maternal cigarette smoking and infant birth weight. Wu and colleagues [26] also found associations between maternal smoking status, polymorphisms of *CYP1A1* and *GSTT1*, and DNA damage. Other studies have confirmed variable DNA damage caused by smoking in genes that regulate genotoxic metabolism [32,33], although not during pregnancy.

Therefore, there are indications that cigarette smoke toxicity depends not only on the intrinsic characteristics of the toxic components in the environmental stress, but also on the individual susceptibility of the subjects exposed to cigarette smoke.

## 4. Epigenetics Effects on Nuclear DNA

Epigenetics provide a window through which we can understand the impact of the environment, nutrition and lifestyle choices on chronic disease susceptibility and risk, since the very early life [34]. Transient and fixed epigenetic modifications occur throughout the life course in response to endogenous and exogenous stimuli that are critical to developmental programs in mutiple tissues and body compartments [35,36]. Therefore, exposures to different environmental agents, *in utero* and early childhood may influence life-long susceptibility to disease via epigenetic alterations.

Epigenetic regulation is achieved by a combination of several molecular machineries, among which there are DNA methylation, histone modification and the noncoding RNA-mediated gene regulation, especially by microRNA (miRNA) [37].

DNA methylation is one of the most widely studied and well-characterized marks of epigenetic regulation and consists of both specific DNA promoter methylation and global DNA methylation in non-coding areas of the genome. Interestingly, both of them are commonly found altered in most types of cancer [38] and emerging evidence indicates that such alterations also occur in other chronic diseases. Understanding epigenetics is likely to lead to the development of biomarkers for early detection of disease risk and public health or therapeutic interventions to prevent chronic disease.

Global DNA methylation has been proposed as an early epigenomic mark in the initial transition from a normal to a diseased cell [39]. Typically DNA methylation occurs in non-coding areas of the genome in normal cells and a loss of methylation in these non-coding areas is associated with normal aging. However, sudden loss of methylation across the genome is notable in the transition from normal to diseased cells in several human diseases and under different environmental stimuli [40].

Global loss of DNA methylation is a complex phenomenon that is not clearly understood. What is known is that huge decrease in methylation of large regions enriched with cytosines (namely global DNA methylation) cannot be due only to changes in the methylation of gene-coding regions, because these account for less than 5% of cytosines found in human DNA. Rather, global DNA hypomethylation is due to changes in the methylation of CpG rich non-coding areas of the genome, such as satellites SAT2 and SAT3, and interspersed repeat sequences such as long interspersed elements (LINEs), short interspersed elements (SINEs) and long terminal containing repeats (LTRs). These changes may well be adaptive responses, evolution-conserved, that maintain homeostasis and assure cell survival in the face of threatening and noxious environmental stimuli.

Because of the increasing evidence that differential DNA epigenome is linked to human disease, several investigators have studied the association between environmental exposure and epigenetic mechanisms such as tobacco smoke exposure and DNA methylation, especially in the early life period.

Terry and colleagues [41] studied the association between smoking exposure and other epidemiologic risk factors across the life course, starting from prenatal age. They measured DNA methylation in white blood cell DNA from a multiethnic cohort in New York City (born between 1959 and 1963) using a (3H)-methyl acceptance assay. The study reported a higher level of DNA methylation associated to maternal smoking during pregnancy. Using multivariable linear models they found associations with increasing birth length, later age at menarche and nulliparity. Later age at first birth was also associated with higher DNA methylation levels in adulthood.

In two different studies [42,43], Breton and colleagues analyzed global and promoter-specific DNA methylation from buccal cells samples in a large children cohort. Surprisingly, they reported that *in utero* smoke exposure is associated with lower levels of global DNA methylation and higher level of methylation of single CpG loci in eight genes analyzed. Interestingly, another independent study confirmed that global DNA methylation in newborns is associated with exposure to prenatal maternal smoking by measuring global DNA methylation through examination of the cord blood serum of full term babies [44]. The study included babies whose mothers were either passive or active smokers and the authors conclude that circulating fetal DNA from cord blood exhibits aberrant methylation in response to environmental exposures. The different techniques and tissues that were examined could explain the differences in the results obtained by Terry in comparison with the latter studies.

Interestingly, several studies correlate *in utero* exposure to tobacco smoke with gene-specific DNA methylation alterations in the placenta. Suter and colleagues observed that maternal tobacco use is associated with alterations in promoter methylation of placental *CYP1A1* gene and that these changes are correlated with *CYP1A1* expression and fetal growth restriction [45]. Using Illumina HG-12 gene transcription with Infinium27K methylation arrays they also detected changes in placental gene expression and DNA methylation associated with maternal tobacco smoke exposure at an epigenome-wide level. Their work suggested that maternal smoking during pregnancy is associated with altered site-specific CpG methylation, which further correlates with significant changes in gene expression in pathways crucial for ensuring proper growth and development, such as the oxidative stress pathways [46]. The strong association between maternal tobacco use and aberrant placental metabolism and multiple markers of oxidative damage has also been demonstrated by Sbrana and colleagues [47].

Interestingly, in a recent genome-wide methylation study conducted on umbilical cord blood samples from a Norwegian Mother and Child Cohort study (MoBa), the association between methylation of *CYP1A1* gene and smoke exposure has been confirmed. Indeed, Joubert and colleagues observed a strong dose dependent association between maternal smoking during pregnancy and the methylation of genes involved in the pathway for the detoxification of xenobiotics in tobacco smoke such as *CYP1A1* and aryl-hydrocarbon receptor repressor (*AHRR*) together with a trascriptional repressor gene growth factor independent 1 transcription repressor (*GFI1*) that is involved in diverse developmental process [48]. Moreover, the differential methylation of *CYP1A1 AHRR* and *GFI1* persist also during adolescence [49].

Additionally, a significant association between *in utero* tobacco exposure and methylation levels of a parentally imprinted gene has been characterized. Indeed, Murphy and colleagues have shown that prenatal exposure to cigarette smoke can cause increased methylation of differentially methylated regions (DMR) in the paternally expressed Insulin-Like Growth Factor II (*IGF2*) gene, from the analysis of umbilical cord blood of 418 pregnant women. They showed that higher methylation of the *IGF2* DMR was associated with low birth weight, predominantly in male infants [50]. *IGF2* plays an important role in regulating growth [51] and is also involved in cancer. This study shows a possible mechanism of action of how smoking influences the growth and development of the fetus.

Exposure to tobacco smoke *in utero* is linked to behavioral and cognitive problems [37]. Some studies report an association between promoter methylation of brain-derived neurotropic factors (*BDNF*) in blood samples from adolescents whose mothers smoked during pregnancy. This gene is important for long-term memory, it helps to support survival of existing neurons and encourages the growth and differentiation of new neurons and synapses [52]. *BDNF* appears to be very susceptible to methylation by environmental factors, especially in exon 6, but other preliminary studies identified methylation changes upstream of exon 1 as potential diagnostic blood biomarkers for major depression [53].

Finally, a very interesting study has been recently published by the group of Wilcox [54] that performed an epigenome-wide association study using whole blood from 889 infants, including 287 whose mothers smoked during the first trimester. They have been searching for multiple CpG sites in or near the same gene and with special focus on possible smoking-associated methylation regions unique in adults or newborns. Intriguingly, they identified 10 new differentially methylated genes in infants whose mothers were smoking subjects, among them genes that are implicated in processes related to nicotine dependence and smoking cessation *(FRMD4A-*FERM domain containing 4A-*ATP9A*-ATPase, class II, type 9A) and placental and embryonic development *(MEG3*-maternally expressed 3). Moreover, the most remarkable observation in their data is the identification of unique smoking-related methylation changes in adults *versus* newborns, suggesting potential age-related differences in the response to exposure. These differences may be related to age-related metabolic and detoxification rates, mediated by epigenetic susceptibility to environmental stress.

Among the large amount of data collected so far, divergent results have been obtained, but this could be a consequence of several limitations intrinsic in each study. For example, some studies are limited by the use of DNA from different tissues or the use of different surrogate methods of quantifying DNA methylation. Other studies are constrained by the method of quantification of the exposure to cigarette smoke. Some studies determine *in utero* smoking exposure by self-report, others use air monitoring of nicotine concentration, or measure the levels of cotinine, a nicotine metabolite, in blood or urine. Finally, other studies are yet limited by not taking into account the impact of second-hand smoke exposure.

The most studied and best-characterized epigenetic mark is 5-methylcytosine (5mC), regulated by the action of various DNA methyltranferases (e.g., *DNMT1, DNMT3A*, and *DNMT3B*). In 2009, two seminal papers described the existence of a new epigenetic modification, 5-hydroxymethylcytosine (5hmC), that was present in high levels in neurons and embryonic stem (ES) cells and arises from the oxidation of 5mC by a group of Fe2+ and 2-oxoglutarate-dependent dioxygenases belonging to the mammalian TET family, namely TET1, 2 and 3 [55,56]. From the analysis of their expression patterns, different functions have been attributed to TET proteins during embryonic and postnatal development [57]. For example, 5hmC is enriched in the paternal genomes [58,59] and 5mC oxidation has been proposed as an important mechanism for erasure of paternal methylation marks [60]. Regarding the biological role of 5hmC, this modification has been found enriched at the borders of the promoters of actively transcribed genes, and proposed as a protection mechanism from DNA methylation spreading [61,62].

Even if a possible role for DNA hydroxymethylation as biosensors of environmental stress could be expected, as a consequence of its important role in the regulation of gene expression in crucial moments of mammalian development, at the moment no study has analysed it. Only recently, a manuscript reported a change in the level of global DNA methylation and hydroxymethylation associated with exposure to urinary cadmium and arsenic [63]. It is more than likely that this could be due to the lack of tools to dissect the hydroxymethylome from the methylome, since the conventional cytosine methylation-profiling methods, including bisulfite sequencing, cannot distinguish between 5mC and 5hmC.

The link between noncoding RNA-mediated gene regulation and maternal cigarette smoking during pregnancy is not well established. MicroRNAs (miRNA) control gene expression by base-pairing to the 3’-untranslated region of the target messenger RNA, which results in problems translating the messenger RNA (mRNA) into a protein molecule, or in degradation of the target mRNA. It is also known that a single miRNA has the capability of regulating a number of genes [64], some of which are involved in regulating vital biological processes, including cell proliferation, differentiation, and apoptosis, or cell death [65,66,67].

Maccani and colleagues conducted a study on placental cell lines, to examine the effects of two components of tobacco smoke (nicotine and benzo(a)pyrene), on four candidate miRNAs expressed in the placenta. They showed downregulation of three distinct miRNAs miR-16, miR-21 and miR-146a, involved in cell cycle regulation, growth, immunomodulation and development in the placenta [68]. In a follow-up study, the same group confirmed the association between cigarette smoking during pregnancy and aberrant miRNA expression in the placenta. While this study was conducted on a limited number of samples and lacks comprehensive data (duration of cigarette smoking, pregnancy, cigarette per day usage, environmental pollutant exposure or second hand/passive cigarette smoke exposure), it represents an important first step in determining the associations between maternal cigarette smoking during pregnancy and aberrant miRNA expression in the placenta [69].

Only a small number of studies have examined the associations between prenatal smoke exposure and histone modifications. A recent study, however, found an association between cigarette smoke exposure during spermatogenesis with abnormal histone-to-protamine transition in human sperm [70]. The exchange of histones with protamines (SP1 and SP2) was significantly higher in male smokers, and the exposure to cigarette smoke was also associated with altered expression of protamines and a high SP1/SP2 ratio.

The consequences of these alterations in the histone/protamine exchange ratio and the SP1/SP2 ratio can lead to infertility and decreased quality of sperm. They also can lead to increased DNA damage that can be transmitted to the fetus [71,72].

As this review highlights, the research community has placed particular focus on investigating the influence of maternal cigarette smoking during pregnancy on DNA methylation patterns. Meanwhile, work on the effects of maternal cigarette smoking on miRNA expression, histone modifications or imprinting during pregnancy has been much less comprehensive. These are important future considerations for investigation in the context of *in utero* smoke exposure [37].

## 5. Mitochondrial DNA

Variation in mtDNA content is another effect deriving from the exposure to cigarette smoking [73]. Due to the error-prone nature of the mitochondrial DNA replication and repair machinery, mtDNA is much more vulnerable to DNA damage than the nuclear genome. Therefore, the exposure to external mutagens or reactive oxygen species (ROS) causes a higher rate of mutations in the mitochondrial genome that accumulate over time and reduce the efficiency of mtDNA repair systems [74,75,76].

The proper functioning of mitochondrial DNA depends on its integrity and the number of copies, suggesting that even slight alterations in mtDNA sequences could have profound effects. For example, mtDNA mutations in respiratory chain genes lead to an increase in ROS.

The malfunction of the mitochondria due to mitochondrial DNA mutations and reduced functionality of the respiratory chain lead to a change in the number of copies of mtDNA and to an increase of mitochondrial mass. The increase of the number of copies of mtDNA, according to some researchers, is considered a compensatory mechanism in respiratory function [77,78,79]. This mechanism of response to the action of genotoxic agents or oxidants, such as smoke, could be an indicator of the level of alteration of mitochondria and a possible mechanism of action of this agent [73,80].

A few studies have examined the relationship between maternal smoking and mitochondrial functionality in humans as well as in animals, and the results are controversial. In humans, studies have reported dose-dependent associations between mtDNA content and smoking in saliva and lung tissue [73,80].

Regarding the prenatal effects, Bouhours-Nouet and colleagues [17] measured the relative mtDNA content and the activity of complex III in placentas from smoking and non-smoking mothers. They report a 29% reduction in the enzymatic activity of complex III, but also a 37% reduction of relative mtDNA content in placental tissue from smokers. Both the enzymatic activity of complex III and mtDNA content were inversely related to the daily consumption of cigarettes. MtDNA content was correlated with cord blood insulin-like growth factor-binding protein-3, a marker of fetal growth that can likely indicate a reduced placental supply of nutrient in smoking-exposed newborns, in agreement with previous studies [81,82,83]. These modifications could alter the placental energy-producing system and contribute to the reduced birth weight in newborns of smoking mothers.

Moreover, maternal smoking during pregnancy is associated with adverse outcomes, among which intrauterine growth retardation (IUGR) is the most frequent complication [84,85,86]. Two studies report increased mtDNA content in placentas and the blood of women carrying IUGR [17,18]. Moreover, they also describe a higher number of mtDNA copies in IUGR placenta samples with a negative correlation to umbilical pO2. The correlation between *in utero* smoke exposure, IUGR and the increase in mtDNA copy number, suggests that cigarette smoke can also affect fetal growth by altering mitochondrial function. Some suggest that the increase in the number of copies of mtDNA is a compensatory mechanism to offset the reduction of mitochondrial functionality [18,87].

In mice exposed to *in utero* or neonatal second hand smoke, Fotterman and colleagues noticed an increase in mtDNA copy number in aortic tissue [88]. Another study conducted on *Maraca mulata* showed a decrease in mtDNA copy number in aortic tissue after perinatal exposure to ETS accompanied by a significant increase in oxidative stress, mitochondrial damage and dysfunction [89].

## 6. Epigenetic Effects on Mitochondrial DNA

Mitochondrial DNA alterations have been proven as an important aspect of mammalian mitochondrial physiology and, similarly to nuclear DNA methylation, abnormal mitochondrial DNA could be the consequence of environmental stress [90]. There are some intrinsic differences between nuclear and mitochondrial DNA that have to be considered when analyzing their respective genetic and epigenetic regulation [91]. Because of this, it is highly likely that a combination of different mitochondria-specific methods should be used to evaluate the extent of mtDNA methylation and test the hypothesis that this modification might follow specific mechanisms different from nuclear DNA epigenetic modifications. Mitochondria certainly contain the machinery required to epigenetically modify mtDNA. Expression studies have demonstrated the activity of mitochondria-localized DNA methyltranferases (*DNMT1, DNMT3BA* and *DNMT3B*) [92] and TET methylcytosine dioxygenases (*TET1, TET2*, and *TET3*) [93].

The role of environmental influences on mtDNA has been the focus of a limited number of studies, but some interesting data has already been collected, such as the discovery of higher methylation levels within the Phe-mtRNA and 12S rRNA coding regions in workers exposed to airborne pollutants [92,93,94]. A recent study also confirmed that cigarette smoke induces methylation and inactivation of the mitochondrial transcription factor A (*mtTFA*) gene promoter, leading to subsequent mitochondrial respiratory complex dysfunction. They also demonstrated for the first time the therapeutic efficacy of treatment with demethylating agents in the reversion of these adverse effects [95].

In contrast with the recent characterization of *DNMT1* in the mitochondrial matrix, bound to mtDNA and modifying transcription of the mitochondrial genome, there is no evidence in the literature regarding a hypothetical function of the *TET* family of methylcytosine oxygenases in the mitochondria. However, both epigenetic modifications (5mC and 5hmC) have been found in mtDNA. *DNMTs* and *TETs* proteins have also been identified in the mitochondria in DNA samples extracted from blood and cultured cells from both humans and mice. In addition, the majority of methylated cytosines in mitochondria are located outside of CpG nucleotides [92,93] Together, this evidence warrants further investigation.

## 7. Pre-Conceptional Exposure

Exposure of maternal and paternal gametes to biological, chemical and physical agents before conception can have profound effects on the newborn. Unfortunately, the association of exposures prior to conception with genomic stability in the offspring is difficult to analyze in human populations, because it can not be separated from gestational exposures. Maternal environmental smoke exposure affects the health of the developing individual and increases the risk of congenital malformations [96]. There are also many epidemiologic studies that report associations between paternal smoking and increases in spontaneous abortions [97] or birth defects [98]. Finally, to highlight even more their importance, the International Agency for Research on Cancer (IARC) has determined that paternal and maternal pre-conceptional exposure and gestational tobacco smoke consumption during pregnancy are linked to an increased risk for childhood cancer in the unexposed newborn [99].

Interestingly, cigarette smoking has been directly linked also to numerical chromosome abnormalities in gametes, which finally determine serious infertility problems, such as pregnancy loss, or developmental disabilities and mental retardation in the progeny [100]. Some studies clearly pointed to smoking as a risk factor for aneuploidy, in particular disomy YY [101], disomy XX [102] and disomy of chromosome 3 [103], indicating that smoke contaminants could cause errors in both meiosis phases I and II. However, other groups obtained different results, for instance, Shi and colleagues haven’t observed an increased risk of chromosome aneuploidy in sperm from smokers [104]. Naccarati and colleagues observed an association between smoking and meiosis II non-disjunction of sex chromosomes [105]. Probably these different conclusions derive from different characteristics of the analyzed cohorts, since many studies have been performed in men undergoing infertility treatments or under multiple exogenous stress.

Other studies have analyzed the consequences of cigarette smoking on the vitality and health of human gametes. For example, sperm form smokers have a higher level of DNA strand breaks [106] and higher DNA fragmentation is seen in spermatozoa from smokers [107]. Smokers have a higher frequency of oocytes with diploid complements of chromosomes when compared to non-smokers [108]. Interestingly, in a case-control study where mothers did not smoke, paternal smoking prior to conception was associated with increased risk of childhood cancer within the child’s first five years [109].

Laubenthal and colleagues analyzed the genomic stability in the offspring exposed to pre-conceptional paternal smoking and gestational maternal smoking, to discriminate between gestational exposure and exposure prior to conception in 39 families [110]. They showed that pre-conceptional paternal smoking and gestational maternal smoking were found to significantly predict DNA damage in the cord blood of F1 offspring. Although on a limited number of samples, this result also supports the claim that cigarette smoke is a human germ cell mutagen because alterations of DNA mutations can be permanent and transmitted to the next generations.

Cigarette smoke metabolites may alter the genomic stability inducing DNA damages such as epigenetic modifications, which can be transmitted to the next generation. Youngson and Holliday demonstrated that cytosine methylation and chromatin structure patterns in spermatozoa were transmissible throughout subsequent generations [111,112]. Methylation plays an important role in the period prior to conception and in early embryonic phase, where the DNA of the parental gametes undergoes the global depletion and subsequent re-establishment of methylation, known as reprogramming. The first reprogramming event occurs at the time of production of the germ line. The primordial germ cells undergo a comprehensive epigenetic erasure that includes imprinted genes. Subsequently, the DNA is de novo methylated and the imprint is re-established [113]. During the pre-implantation development period, the second reprogramming occurs and this allows the embryonic stem cells to become totipotent. Only the imprinted genes do not undergo this change to preserve parent-of origin expression [114]. The imprinted genes are expressed in a parent-of-origin-specific manner, and they have roles in the control of embryonic growth and development. Lane and colleagues reported two DNA regions resistant to DNA methylation reprogramming, the IAPs and LTR-ERV1 elements [115,116].

Germ-line and post fertilization reprogramming are two extremely important and vulnerable phases for the development of the fetus [117]. If reprogramming fails, if deleterious epigenetic mutations occur on imprinted genes, or if there is a non-maintenance of protected areas, the damage may persist and be transmitted to the offspring [113,118].

Environmental factors, even in the absence of continued exposure, may affect DNA reprogramming, persist through next generations and compromise their health [12] defining trans-generational epigenetic inheritance [113]. Studies on animals show that paternal insults prior to conception due to exposure to toxins [12], nutrition [119] or ionizing radiation [120] on animals can affect the health of the offspring. In addition, aberrant methylation on imprinted genes is associated with infertility and chronic disorders in the offspring [121,122].

Therefore, it can be assumed that environmental factors such as cigarette smoking may have the same effect, especially if we consider that cigarette smoking is one of the most powerful agents capable of modifying DNA methylation [123]. However, further studies are required, to shed light on this point.

Moreover, hypothetically, epigenetic and DNA damages in nuclear DNA can also influence the regulation of genes responsible for maintaining genome stability and alter regular DNA damage response and repair processes. This hypothesis is a possible explanation for the elevated strand breaks found in cord blood DNA from newborns exposed to cigarette smoke *in utero* [124,125].

## 8. Conclusions

Tobacco smoke ranks as an important public health problem whose adverse impacts have spread around the world. The concern increases if we consider the association between exposure to prenatal smoke and increased risk of spontaneous abortion, preterm delivery, respiratory disease, immune system difficulties such as asthma and allergies, placental complications and increased risk of illnesses, health problems, cancer and psychological outcomes later in development [4,126,127]. The mechanisms by which tobacco smoke causes adverse health effects in the newborn have been studied intensively during the past 50 years. Based on current knowledge, multiple mechanisms take action in different periods of fetal development, from pre-conception to the perinatal period. The genotoxic and mutagenic potential of cigarette smoke can lead to damage in both nuclear and mitochondrial DNA, resulting in the observed effects reported in the literature. We have reviewed the literature that focuses on genetic and epigenetic effects on nuclear and mitochondrial DNA.

Most of the research about the health effects of cigarette smoke have focused on mutagenic and genotoxic effects on nuclear DNA, but more recently, epigenetic studies have shown that cigarette smoke can influence the parental, fetal, or placental epigenome, altering gene regulation mediated by DNA methylation, histone modifications and noncoding RNAs. Genetic and epigenetic effects of cigarette smoke could contribute together to compromise the health of the individual but, the relationships between them still needs to be better understood. However, the literature reports that cigarette smoking acts mainly on some target regions on DNA, such as genes of the chemical detoxification pathway.

As explained before, the genotoxic potential of tobacco compounds require a metabolic activation by Phase I detoxification enzymes, while Phase II enzymes, catalyze the activation or deactivation of aromatic and heterocyclic amine carcinogens. *CYP1A1* is a gene coding for a Phase I enzyme and is often reported in the literature in relationship to cigarette smoke [27,45]. In fact, polymorphisms of *CYP1A1* affect susceptibility to tobacco smoke [27]. Furthermore, cigarette smoke alters *CYP1A1* site-specific CpG methylation and gene expression [46,47]. *CYP1A1* in particular, but also other genes involved in chemical detoxification pathway, may represent a specific target for tobacco smoke, which can modify its function in a genotoxic or epigenetic way.

Importantly, cigarette smoke contains and generates various ROS, which can damage nuclear DNA but also mtDNA. Recently, many groups have noted variation in mtDNA copies number, in response to damage to mtDNA and the respiratory chain, associated to cigarettes smoke exposure [73,128,129]. Interestingly, variations in mitochondrial DNA copy number and damages to the respiratory chain in the placenta and cord blood have been linked to adverse outcomes in newborns [17,80,81,82,83,84,85].

Mitochondrial DNA is extremely vulnerable. Defects in the respiratory chain can lead to an increased production of ROS, increasing in turn the probability of nuclear DNA damage and increases in mtDNA content, a compensatory mechanism for the damages to the respiratory chain and the lack of energy production [79,80]. Moreover, genes belonging to oxidative phosphorylation and mitochondrial dysfunction pathways are differentially methylated among smokers [46,47].

In conclusion, cigarette smoke leads to adverse effects on nuclear DNA and mtDNA, affecting the health of newborns by compromising their energy intake. Furthermore, since mtDNA is maternally inherited, it is conceivable that maternal exposure to cigarettes smoke prior to conception can lead to mtDNA alterations, which are subsequently transmitted to new generations. More investigations on humans will be necessary to clarify the influences of cigarette smoke on mtDNA and the interactions with genomic DNA alterations, opening another chapter in the understanding of cigarette smoke mechanisms of action.

In addition to providing an overview on the mechanisms of action of cigarette smoke adverse effects on nuclear and mtDNA, this review wants to emphasize the importance of parental life-styles in the future health of their unborn children. As mentioned before, there are three sensitive periods during the very early stage of development where a disruption or modification of the environment can influence fetal and offspring development: preconception, *in utero* and perinatal. Not much attention has been placed on the preconception period, mainly due to the difficulty of distinguishing pre-conceptional effects to those that occur in the two later periods. However, the literature reports chromosomal aberrations and DNA damages in parental gametes, in particular in sperm [100,101,102,103,104,105,106,107,108,109] as well as in newborns’ genomes [21,23,25,27]. Moreover, the discovery of epigenetic inheritance reinforces the importance of exposures prior to conception, as a crucial period for the predisposition to chronic diseases and cancer [118]. More studies are needed to expand our knowledge of the epigenetic effects of cigarette smoke on gametes, imprinted genes, and how they are transmitted to the next generation. In sum, a deeper awareness of the contribution of exposures prior to conception will shift the focus of the discussion and may lead to increased prevention of harmful life-style habits during the life-course, not just during the *in utero* and early life periods.
